# Immunoglobulin G4-Related Disease-Associated Dermatitis with Pruritus: A Positive Response to Dupilumab

**DOI:** 10.3390/life13030833

**Published:** 2023-03-20

**Authors:** Tyler C. Beck, John Plante, India Robinson, Katsiaryna Khatskevich, Jessica A. Forcucci, Manuel Valdebran

**Affiliations:** 1 Department of Dermatology and Dermatologic Surgery, Medical University of South Carolina, Charleston, SC 29425, USA; 2 Department of Pathology and Laboratory Medicine, Medical University of South Carolina, Charleston, SC 29425, USA; 3 Department of Pediatrics, Medical University of South Carolina, Charleston, SC 29425, USA

**Keywords:** dupilumab, biologic, dermatitis, lichen amyloidosis, primary cutaneous amyloidosis, primary localized cutaneous amyloidosis, IgG4-related disease, IgG4-related skin disease, immunoglobulin G4-related disease, immunoglobulin G4-related skin disease

## Abstract

Immunoglobulin G4-related disease (IgG4-RD) is a rare fibro-inflammatory condition characterized by IgG4-expressing plasma cell infiltration of the skin and other organs, leading to profound itchiness. Oral corticosteroids are the first-line therapy for IgG4-RD but relapses and potential side effects are common. In this case, we discuss a patient with a hyperpigmented, scaling dermatitis on his arms, back, and chest with lichen amyloidosis (LA) that incompletely responded to corticosteroids. He had reduced quality of life secondary to chronic pruritus. Dupilumab, an IL-4 and IL-13 inhibitor, was initiated. He experienced a transient worsening, followed by complete resolution of his itch with remission of his rash. While the pathogenesis of IgG4-RD is not entirely understood, a T-helper 2 (Th2) immune response has been implicated, with interleukins (IL) 4, 5, 10, and 13 playing a role in IgG4 class switch, resulting in eosinophilia and elevated IgE. The strong response of dupilumab in this case may provide evidence in favor of the involvement of IL-4 and IL-13 in the pathogenesis of cutaneous IgG4-RD. Future clinical studies involving larger patient populations may be warranted.

## 1. Introduction

Immunoglobulin G4-related disease (IgG4-RD) is a rare fibro-inflammatory condition characterized by IgG4-expressing plasma cell infiltration of the skin and other organs [1]. IgG4-RD typically affects middle-aged or elderly males and can present with widespread cutaneous and systemic manifestations [2,3,4,5]. Cutaneous IgG4-RD exhibits a heterogenous clinical picture but commonly presents alongside internal organ involvement as erythematous and pruritic papules, nodules, or plaques on the head and neck or trunk. Oral corticosteroids are first-line therapy for IgG4-RD. However, relapses are common and potential side effects are multiple [6]. Here we present the unique case of IgG4-related disease associated dermatitis and pruritus (IgG4-ADP) with lichen amyloidosis (LA); a condition that is often secondary to chronic pruritus [7,8,9,10,11]. The patient did not tolerate rituximab and had a variable response to corticosteroids but demonstrated a complete response to dupilumab.

## 2. Case Report

Our patient is a Middle Eastern male with a past medical history of asthma and allergic rhinitis. In 1989, he presented at 36 years of age to an outside institution with sclerosing cholangitis and pancreatitis. He was diagnosed with IgG4-RD after serologic evaluation and a strong response to steroids. Over the next several decades, he experienced intermittent disease flaring on low–moderate dose prednisone. Of note, in 2016, he was diagnosed with stage IV chronic kidney disease secondary to systemic IgG4-RD; however, his renal function remained unchanged over the course of this case report (Table A1 in Appendix A).

He first presented to our institution in 2016 with fatigue and elevated blood urea nitrogen (BUN) (68 mg/dL) (normal range (NR): 5–25 mg/dL) and serum creatinine (2.8) (NR: 0.6–1.3 mg/dL), as well as a low estimated glomerular filtration rate (eGFR) of 23. His liver function tests (LFTs) were within normal reference ranges. A renal biopsy demonstrating severe interstitial nephritis with fibrosis was suspected by nephrology to be IgG4-related tubulointerstitial nephritis. At that time, he was diagnosed with stage IV chronic kidney disease (CKD). He quickly responded to high-dose steroids and was discharged on 20 mg/day of prednisone.

During a rheumatology appointment in late 2016, the patient reported fatigue and severe, progressive pruritus over the past several months, interfering greatly with his quality of life. A diffuse, erythematous, confluent papular rash was observed on his back and arms. Dry eyes and mouth were also present, and his serum IgG was 506 mg/dL (normal range (NR): 1–123 mg/dL). During this visit, the patient reported that a rash and severe, diffuse pruritus had started on his back and arms several years ago and was refractory to hydroxyzine, famotidine, moisturizing creams, dapsone cream, and topical corticosteroids. A prior biopsy showed interface dermatitis and perivascular inflammation with eosinophils. 

**Figure 1:**
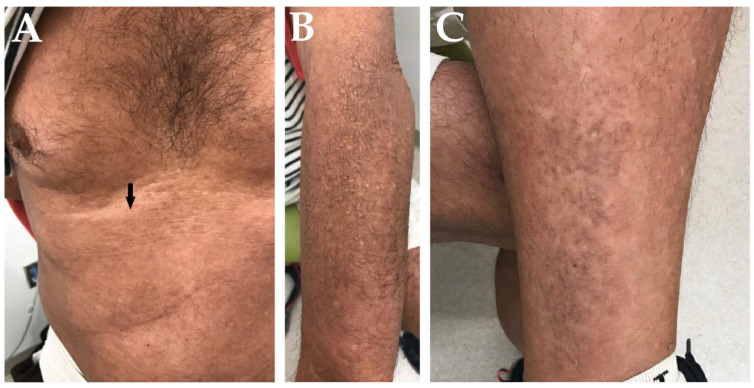
(**A**) Immunoglobulin G4-related disease-associated dermatitis involving the abdomen (arrow). Lichen amyloidosis involving the left forearm (**B**) and left leg (**C**) prior to initiating dupilumab therapy.

Steroid-sparing therapy with rituximab was initiated at the end of 2016. His rash and pruritus quickly improved, and his symptoms remained well-controlled after a steroid taper until November of 2019. His IgG4 level at that time was within the normal range (10 mg/dL). His pruritus later returned, and a hyperpigmented, scaling rash on his arms, back, and chest was identified. Rheumatology then placed a dermatology referral. Rituximab was continued through the middle of 2020 until he was hospitalized for suspected rituximab-induced neutropenia. He was discharged on 10 mg/day of prednisone without rituximab. His pruritus worsened over the next several months despite the addition of topical corticosteroids, and he presented to our dermatology clinic toward the end of 2020. His exam was remarkable for ill-defined, erythematous plaques on his abdomen (Figure 1A) and dark tan papules on bilateral forearms and left leg (Figure 1B,C). 

**Figure 2:**
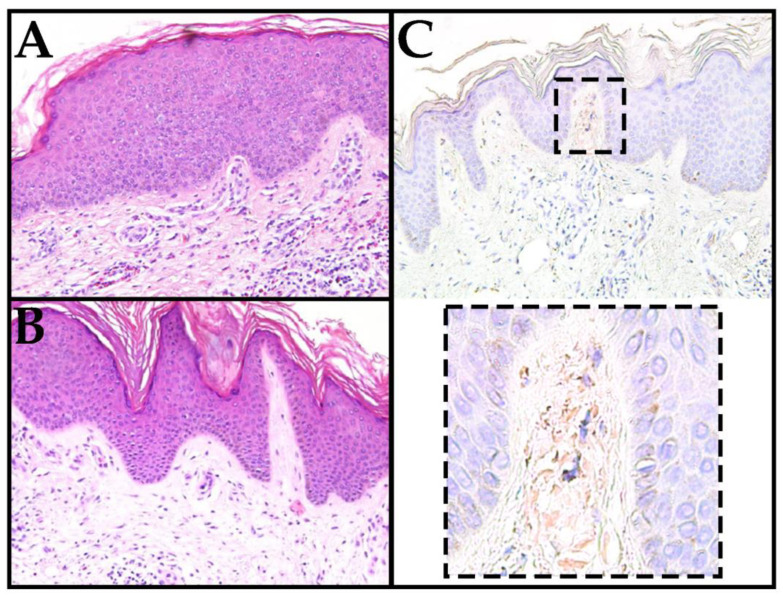
(**A**) A shave biopsy from the patient’s forearm showed spongiotic dermatitis with mixed superficial perivascular inflammation with notable eosinophils (H&E 200×). (**B**) The biopsy revealed lichen amyloidosis (H&E 200×). (**C**) Congo Red staining highlighted small amyloid deposits in the superficial papillary dermis (200× with digital zoom).

Lab work was significant for peripheral eosinophilia, and a workup resulted in the following: a positive antinuclear antibodies test (1:80 nucleolar pattern); elevated absolute eosinophil count (AEC) (2820 cells/mcL) (NR: 30–350 cells/mcL); elevated IgE (281 IU/mL) (NR:1.5–144 IU/mL); normal adrenocorticotropic hormone, cortisol, and tryptase levels; and negative strongyloides and toxocara antibodies. A tangential biopsy of his abdomen demonstrated spongiotic dermatitis with mixed superficial and perivascular infiltrates with eosinophils (Figure 2A). An incisional biopsy of his right forearm showed small deposits of amyloid in the superficial papillary dermis, and the diagnosis of LA was made (Figure 2B,C). Subepidermal deposition of amyloid correlates to keratinocyte-derived amyloid rather than a systemic origin.

Given the constellation of conditions contributing to his pruritus, such as IgG4-RD, LA, and atopy, a course of dupilumab therapy was initiated at 600 mg as a loading dose followed by 300 mg biweekly injections. Worsening pruritus and erythema were reported after one month, and his AEC increased to 3610 cells/mcL. During this time, no changes in liver or renal function were observed and no new medications were initiated. One month later, his pruritus had significantly improved spontaneously, and his AEC was 580 cells/mcL. Three months after starting dupilumab, he noted further improvement in his pruritus, and his prednisone was tapered to 5 mg/day. His AEC was 220 cells/mcL, and his IgG was 382 mg/dL.

**Figure 3:**
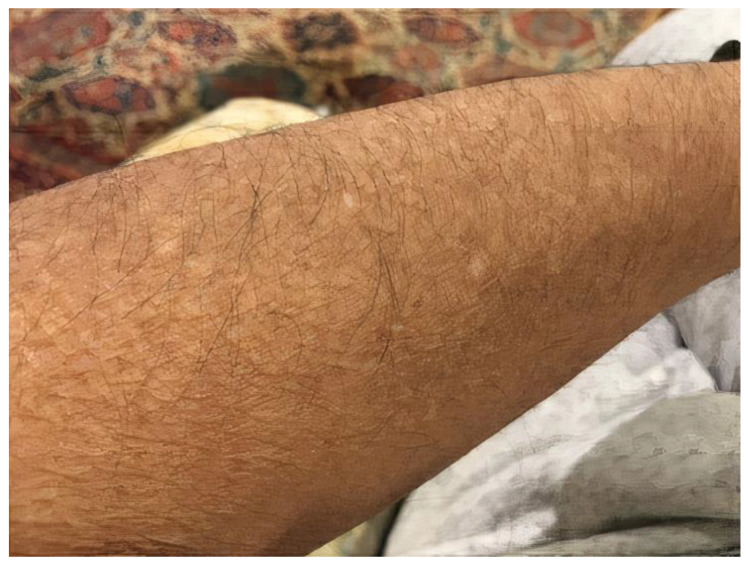
Left forearm demonstrating resolution of hyperpigmented papules after 4 months of dupilumab therapy.

At his 4-month follow-up appointment in early 2021, he reported a remarkable improvement in his quality of life. His LA demonstrated significant improvement (Figure 3), and his AEC had further decreased to 90. His pruritus is still well-controlled on dupilumab after two years of treatment. His most recent labs revealed an AEC of 160 cells/mcL, IgE of 141 IU/mL, and C3 of 85 mg/dL (NR: 88–201). At his most recent follow-up in the spring of 2022 his eosinophil and neutrophil counts remained normalized, and he remained in remission from his dermatitis and pruritus. 

## 3. Discussion

Given our patient’s clinicopathologic features and lab results, including peripheral eosinophilia and elevated IgE, we concluded his cutaneous findings and pruritus were compatible with a cutaneous manifestation of IgG4-RD that we termed IgG4-ADP [2,12]. Attempts to control his IgG4-RD, LA, and atopy with corticosteroids and anti-histamines were unsuccessful. As such, novel approaches to treat his disease were initiated. 

While the pathogenesis of IgG4-RD is not entirely understood, a T-helper 2 (Th2) immune response has been implicated, with interleukins (IL) 4, 5, 10, and 13 playing a role in IgG4 class switch, resulting in eosinophilia and elevated IgE. Dupilumab is an IL-4 and IL-13 antagonist that potentially inhibits Th2 IL production. In addition, dupilumab may inhibit IL-31 production, a cytokine implicated in pruritus [10]. This is a plausible explanation for the strong response of our patient’s pruritic cutaneous disease to dupilumab.

We report a case where dermatitis, itch, and peripheral eosinophilia were resolved with dupilumab treatment, with a significant improvement in quality of life sustained two years post-treatment initiation. This report provides additional insight into the pathogenesis of these diseases and further supports the efficacy of dupilumab in itch-related processes. Our patient experienced dramatically improved quality of life with initiation of therapy. Awareness of cytokines activated by IgG4 and how they manifest clinically and in ancillary studies is important for targeted therapy. Future clinical study of dupilumab and its use in the treatment of both systemic and cutaneous IgG4-RD in larger patient populations should be considered.

## 4. Limitations

This case report was not without limitations. Although skin biopsies cannot directly prove lesions are connected, the constellation of findings such as elevated eosinophils leading to pruritus and spongiotic dermatitis, as well as response to treatment, demonstrate evidence of a connection to IgG4-RD.

## Data Availability

No new data were generated in the creation of this case report.

## Appendix A

**Table A1:** Lab Values Assessing Renal and Liver Function.

	4/16	1/17	11/19	6/20	8/20	11/20	1/21	3/21	9/21	2/22	Units
**BUN**	68	61	51	45	79	45	48	41	43	47	mg/dL
**CR**	2.8	3.1	2.4	2.1	3.1	2.7	2.6	2.7	2.8	2.7	mg/dL
**eGFR**	23.0	20.0	27.0	32.0	20.0	23.5	24.6	23.5	22.3	23.3	mL/min/1.73 sqm
**Bili (Total)**	0.5	0.6	0.8	0.8	0.6	0.7	0.8	0.8	0.9	1.1	mg/dL
**AST**	24	23	27	19	31	21	14	20	21	21	U/L
**ALT**	35	31	25	24	42	24	17	20	20	20	U/L
**ALP**	88	84	86	82	69	84	93	81	116	119	U/L

Lab values remained relatively unchanged over the course of the case report. The patient demonstrated poor renal function at baseline, which remained consistent over the course of his treatment. Abbreviations: BUN (blood urea nitrogen); CR (creatinine); eGFR (estimated glomerular filtration rate); Bili (Total) (total bilirubin); AST (aspartate transaminase); ALT (alanine transaminase); ALP (alkaline phosphatase). Normal ranges: BUN (5–25 mg/dL); CR (0.6–1.3 mg/dL); eGFR (>60 mL/min/1.73 sqm); Bili (Total) (0.0–1.0 mg/dL); AST (14–50 U/L); ALT (10–40 U/L); ALP (50–136 U/L).
